# Genome-wide characterization and identification of candidate *ERF* genes involved in various abiotic stress responses in sesame (*Sesamum indicum* L.)

**DOI:** 10.1186/s12870-022-03632-7

**Published:** 2022-05-24

**Authors:** Ruqi Su, Senouwa Segla Koffi Dossou, Komivi Dossa, Rong Zhou, Aili Liu, Yanping Zhong, Sheng Fang, Xiurong Zhang, Ziming Wu, Jun You

**Affiliations:** 1 Key Laboratory of Biology and Genetic Improvement of Oil Crops of the Ministry of Agriculture and Rural Affairs, Oil Crops Research Institute of the Chinese Academy of Agricultural Sciences, Wuhan, 430062 China; 2 Oil Crops Research Institute of the Chinese Academy of Agricultural Sciences, Key Laboratory of Biology and Genetic Improvement of Oil Crops of the Ministry of Agriculture, Wuhan, 430062 China; 3 CIRAD, UMR AGAP Institut, F-34398 Montpellier, France; 4 UMR AGAP Institut, Univ Montpellier, CIRAD, INRAE, Institut Agro, F-34398 Montpellier, France

**Keywords:** *ERF* gene family, *Sesamum indicum*, Transcription factors, Gene expression, Abiotic stress

## Abstract

**Background:**

The adverse effects of climate change on crop production are constraining breeders to develop high-quality environmentally stable varieties. Hence, efforts are being made to identify key genes that could be targeted for enhancing crop tolerance to environmental stresses. ERF transcription factors play an important role in various abiotic stresses in plants. However, the roles of the ERF family in abiotic stresses tolerance are still largely unknown in sesame, the “queen” of oilseed crops.

**Results:**

In total, 114 sesame *ERF* genes (*SiERFs)* were identified and characterized. 96.49% of the *SiERFs* were distributed unevenly on the 16 linkage groups of the sesame genome. The phylogenetic analysis with the *Arabidopsis* ERFs (*AtERFs*) subdivided *SiERF* subfamily proteins into 11 subgroups (Groups I to X; and VI-L). Genes in the same subgroup exhibited similar structure and conserved motifs. Evolutionary analysis showed that the expansion of *ERF* genes in sesame was mainly induced by whole-genome duplication events. Moreover, *cis*-acting elements analysis showed that *SiERFs* are mostly involved in environmental responses. Gene expression profiles analysis revealed that 59 and 26 *SiERFs* are highly stimulated under drought and waterlogging stress, respectively. In addition, qRT-PCR analyses indicated that most of *SiERFs* are also significantly up-regulated under osmotic, submerge, ABA, and ACC stresses. Among them, *SiERF23* and *SiERF54* were the most induced by both the abiotic stresses, suggesting their potential for targeted improvement of sesame response to multiple abiotic stresses.

**Conclusion:**

This study provides a comprehensive understanding of the structure, classification, evolution, and abiotic stresses response of *ERF* genes in sesame. Moreover, it offers valuable gene resources for functional characterization towards enhancing sesame tolerance to multiple abiotic stresses.

**Supplementary Information:**

The online version contains supplementary material available at 10.1186/s12870-022-03632-7.

## Background

Sesame (*Sesamum indicum* L*.*) is a worldwide important oilseed crop cultivated mainly in tropical and subtropical regions and providing humans with high-quality nutrients and nutraceuticals [1–3]. It represents a priceless material for food, cosmetics, and medicine [4]. For instance, its lignans have been reported to possess various physiological properties, such as antioxidant, antiaging, serum lipid-lowering, blood pressure-lowering, anti-cancer, etc. [5–7]. Therefore, the global market of sesame products is being expanded. Unfortunately, sesame productivity, yield, and seed quality are influenced by several abiotic stresses, including drought, waterlogging, salt, and heat [8, 9]. Among them, drought and waterlogging are the leading environmental adverse impairing physiological and biochemical processes in sesame [10–12]. Studies revealed that plants initiate a series of transcription factors (TFs) phosphorylation/dephosphorylation under stress to enable them to bind *cis*-elements of stress-related genes to enhance or suppress their transcription, thus inducing stress tolerance [13, 14]. TFs are critical in regulating plant’s defense responses to stresses and are emerging as promising resources for engineering improved crop varieties with tolerance for multiple abiotic stresses [15]. In sesame, studies carried out by Dossa et al., and Wang et al. disclosed that ERF, MYB, bHLH, and WRKY TF families are the main genes involved in sesame responses to abiotic stresses [16, 17]. MYB and WRKY TFs have been widely identified in sesame, and their expression under various abiotic stresses was evaluated [18, 19]. However, the ERF gene family is not well characterized in sesame, and only DREB genes expression under drought stress was investigated [20].

ERF, together with AP2 (APETALA2), DREB (dehydration responsive element binding), RAV (related to ABI3/VP), and Soloist (specific proteins) genes are members of the AP2/ERF TFs superfamily [21, 22]. The ERF gene family includes ERF and DREB genes and encodes a protein with a single AP2/ERF domain [23]. The structure of the domain is unique, with three-stranded β-sheets and an α-helix consisting of approximately sixty conserved amino acids [24]. ERF and DREB genes could be distinguished by their DNA binding domains [21]. The ERF subfamily binds to the AGCCGCC of GCC-box, while the DREB subfamily usually interacts with the CCGAC core sequence. ERF TFs are widespread in plants, and numerous ERF genes have been successfully identified in crops, including *Arabidopsis* [22], rice [25], soybean [26], tomato [27], peanuts [28], *Zea mays* [29], *Brassica napus* [30], and wheat [31]. Their roles in plants’ response to abiotic stresses have been extensively studied [32]. For example, *AtERF1* is reported to play a positive role in salt, drought, and heat stress tolerance by regulating stress-specific genes in *Arabidopsis* [33]. Overexpression of *AtERF019* delayed *Arabidopsis* plant growth and senescence and improved drought tolerance [34]. Overexpression of *AtERF71* enhanced the *Arabidopsis* plant tolerance to salt stress and its ability to resist osmotic stress [35]. *AtERF98* enhanced tolerance to salt through the transcriptional activation of ascorbic acid synthesis [36]. In rice, it was demonstrated that *OsERF71* increases the plant tolerance to drought by binding to the promoter of *OsCC1* [37]. Conversely, overexpression of *OsERF922* impaired the plant tolerance to salt stress [38]. In soybean, *GmERF3* was reported to be essential for plant survival under salinity and drought [39]. In cotton, *GhERF38* is essential for the plant response to salt and drought stresses [40].

In the present, the ERF gene family was re-identified in sesame under stringent conditions. Through a comprehensive bioinformatic analysis, their structure, chromosomal distribution and duplication events, phylogeny, and conserved motifs were revealed. Moreover, their expression patterns in response to drought, waterlogging, osmotic, submerge, ABA, and ACC treatments were analyzed. Our findings provide new insights into the ERF gene family and reveal key *SiERF* genes for targeted improvement of the sesame tolerance to abiotic stresses.

## Results

### Genome-wide identification of ERF family genes in sesame

In total, 114 putative ERF genes were identified and named from *SiERF1* to *SiERF114* based on their appearance on the sesame linkage groups. Detailed information of *SiERFs* such as gene name, gene ID, mRNA accession, protein accession, linkage group, gene start position, gene end position, protein length, and the number of exons are shown in Table S1. 

The proteins of the 114 *SiERF* ranged from 121 (*SiERF091*) to 419 (*SiERF114*) amino acids (aa) in length. The molecular weights (MWs) and the isoelectric points (pIs) of the sesame ERF proteins varied from 13.42804 (*SiERF114*) to 46.17756 kDa (*SiERF091*) and 4.5 (*SiERF072*) to 10.24 (*SiERF114*), respectively. Table S2 presents detailed information about the physiochemical proprieties of each identified ERF protein.

### Chromosomal localization and gene duplication analysis of *SiERF* genes

**Fig. 1:**
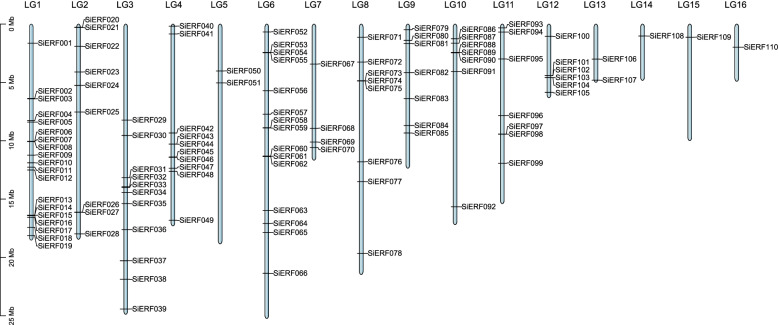
Distribution of *SiERF* genes within the sesame linkage groups (LGs). Vertical bars represent the LGs within the sesame genome. The LG number is indicated at the top of each LG. The scale on the left is in megabases

96.49% of the *SiERF* genes (110 genes) were distributed unequally on the 16 linkage groups (LGs) (Fig. 1). The remaining four *SiERF* genes (*SiERF*111, 112, 113, and 114) are located on the unanchored scaffolds (Table S1). The LG1 harbored the largest number of 19 *SiERF* genes, accounting for 16.67% of the total number. In contrast, the LG14, LG15, and LG16 contained only one *SiERF* gene, respectively. Some *SiERF* genes formed one, two or three clusters on LG1、LG2、LG3、LG4、LG6、LG8、LG10、LG11 and LG12.

In order to reveal the evolution mechanism of the ERF gene family in sesame, we analyzed the duplication events. The result indicated that the *SiERF* gene family underwent whole-genome duplication (WGD) and tandem duplication events (Fig. S1). Fifty-eight (58) *SiERF* genes accounting for 52.73% were derived from WGD events, indicating that whole-genome duplication plays a major role in ERF gene family expansion in sesame. The tandem gene duplication involved 18 *SiERF* genes.

### Phylogenetic analysis among the *Arabidopsis* and sesame ERFs

**Fig. 2:**
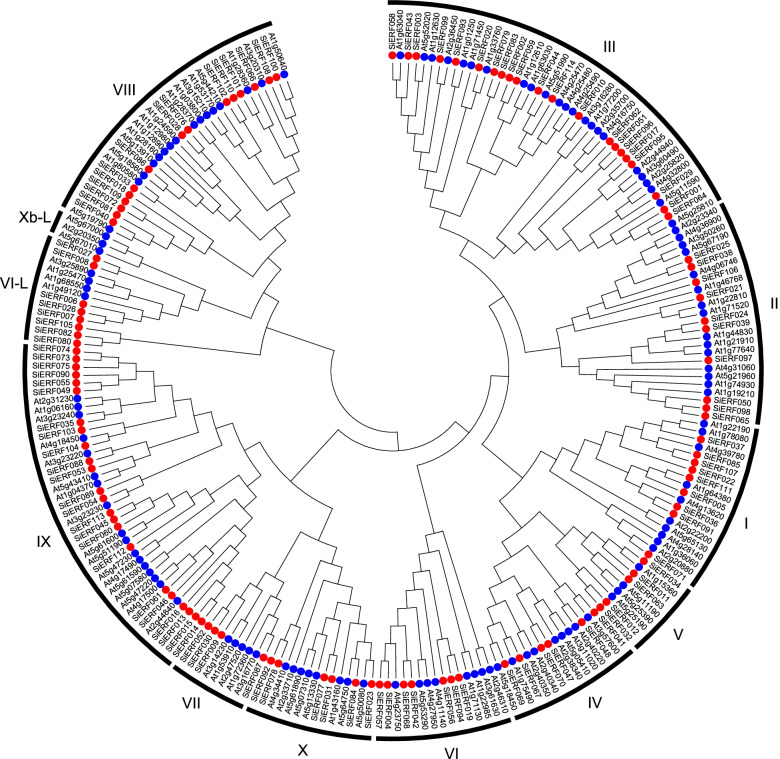
Phylogenetic analysis of the ERF proteins in sesame and *Arabidopsis*. Multiple sequence alignments of ERF amino-acid sequences were conducted using ClustalX, and the phylogenetic tree was constructed using MEGA5 by the neighbor-joining (NJ) method and 1000 bootstrap replicates. The blue triangles and red dots represent ERF proteins in *Arabidopsis* and sesame, respectively

To get insight into the phylogenetic relationships of the ERF gene families, a phylogenetic tree was constructed using the neighbor-joining (NJ) method and based on AP2/ERF domain of 122 *Arabidopsis* ERFs and the 114 *SiERFs*. As presented in Fig. 2, the *SiERFs* were distinctly divided into eleven (11) groups (groups I, II, III, IV, V, VI, VII, VIII, IX, X, and VI-L), which closely agrees with the phylogenetic analysis of ERFs in cassava and *Andrographis paniculate* [41, 42]*.* One additional group (group Xb-L) was composed uniquely of three *Arabidopsis* ERFs. Groups I ~ X and VI-L constituted of 9, 10, 21, 6, 5, 7, 4, 15, 23, 6, and 8 *SiERFs*, respectively. The largest group (class III) included 45 ERF proteins (21 *SiERFs* and 24 *AtERFs*), suggesting that genes of this subfamily might undergo duplication events and retain more genes.

### Gene structure, conserved domain, and *cis*-acting elements analyses of *SiERF* genes

**Fig. 3:**
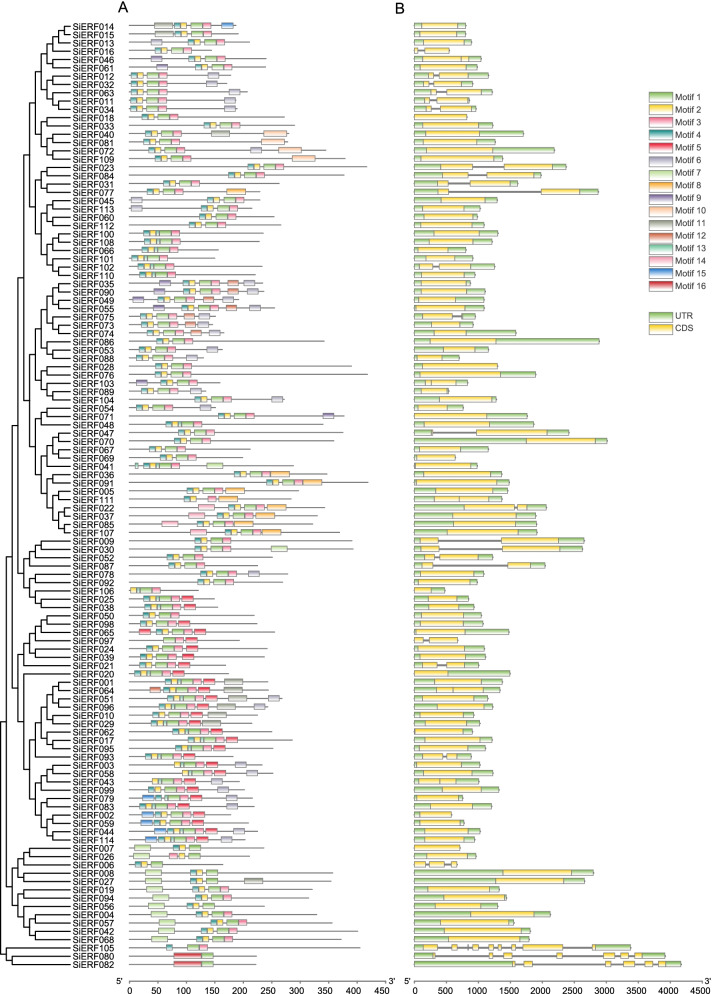
Phylogenetic relationships, gene structure, and motif compositions of *SiERFs*. **A** Left: the phylogenetic tree of Si*ERF*s. Right: conserved motif composition of the *SiERFs*. Different colored boxes represent different motifs. **B** Intron-Exon structure of *SiERFs*. The green boxes represent UTR, grey lines represent introns, and yellow boxes represent exons

Phylogenetic evolution and gene structure usually have a strong correlation. To study the structural characteristics of the *SiERF* genes, the conserved motifs and the number of exons and introns were identified and analyzed. Totally, we identified 16 conserved motifs (motif 1–16) through MEME motif detection software (Fig. 3A). The motifs were constituted of 6 to 49 aa (Fig. S2). Each *SiERF* contained two to eight motifs. The motifs 1, 2, 3, and 4 aligned in the order 4–2–1-3 were shared by 95 *SiERFs*, indicating that ERF family genes are relatively conserved in sesame. Motifs 5 and 13 were shared by 28 *SiERFs*, and motif 6 was shared by 29 SiERFs. *SiERF* proteins in the same group displayed similar conserved motif types (Fig. 3A). For instance, 20, 17, and 13 *SiERFs* in the same groups shared motif 8, motif 7, and motif 11, respectively, indicating that subgroups of *SiERF* are different. To determine the number and location of exons and introns, the structure of *SiERF* genes was further analyzed via the TBtools software. The result showed a weak variation of the number of exons and introns in the sesame ERF gene family (Fig. 3B). 90 of the 114 (78.9%) sesame ERF genes contained only one exon and no intron. Twenty (17.5%) *SiERF* genes contained two exons and one intron.

To identify the putative *cis*-acting regulatory elements in the promoter regions of the *SiERFs*, the sequences 1500-bp upstream from the protein start codons (ATG) of each gene were analyzed by the PLACE database [43]. All *SiERFs* contained *cis*-acting elements within the analyzed interval. Totally, 40 *cis*-elements mainly related to hormone response, stress response, and light-response were identified (Table S3; Table S4). Light responsive elements, including I-box, TCT-motif, TCA-element, TCCC-motif, GT1-motif, GA-motif, G-Box, AE-box, Box 4, MRE, etc., were the most abundant (Fig. S3). Hypoxia response elements (ARE), ABA response elements (ABRE), methyl jasmonate response elements (CGTCA-motif and TGACG-motif), and ethylene response elements (ERE) were detected in 82, 89, 67, 67, and 72 genes, respectively (Table S3).

### Expression profiles of *SiERF* genes under drought and waterlogging stresses

**Fig. 4:**
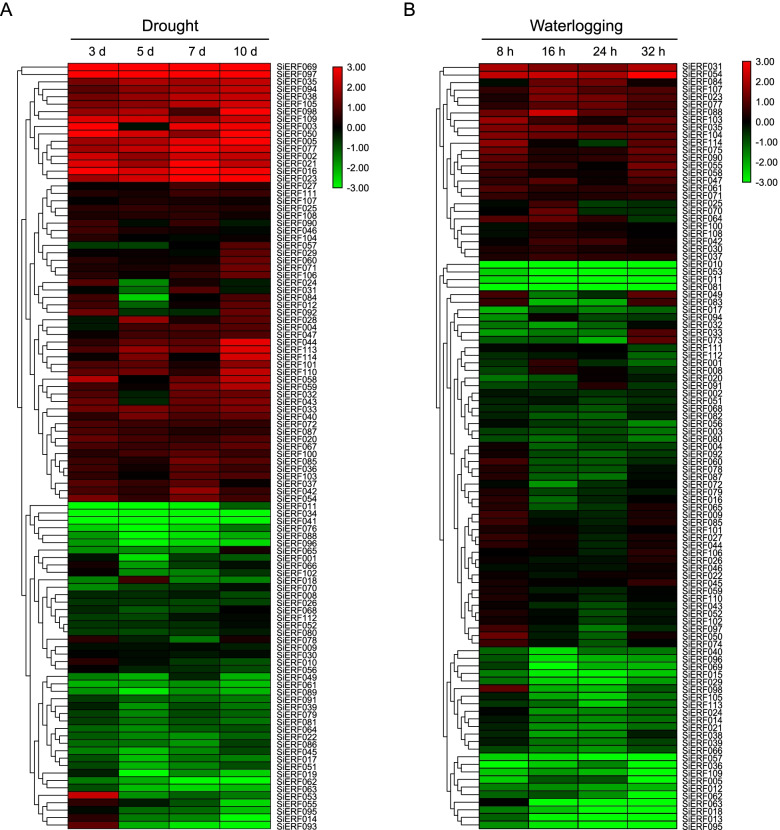
*SiERF* genes expression profiles in sesame roots under drought stress (**A**) and waterlogging stress (**B**). Transcriptome data (Reads Per Kilobase per Million mapped reads; RPKM) were used for the expression levels measurement. The colored scale for the different expression levels is shown

To explore the roles of *SiERF* genes in sesame response to drought and waterlogging stresses, we investigated their expression in roots at different time points based on RNA-seq data from previous studies [9, 44]. Unfortunately, eleven (*SiERF006*, *007*, *013*, *016*, *048*, *073*, *074*, *075*, *082*, *083,* and *099*) and thirteen genes (*SiERF006*, *007*, *019*, *028*, *034*, *041*, *048*, *067*, *076*, *086*, *089*, *093,* and *099*) lacked RNA-Seq data under progressive drought and waterlogging stress, respectively. As shown in Fig. 4A, the *SiERF* genes exhibited significant transcriptional changes in responses to drought stress. 59 (51.8%) and 44 (38.6%) *SiERF* genes were up-regulated and down-regulated under drought stress, respectively. Among the up-regulated *SiERFs*, fifteen (*SiERF002*, *005*, *016*, *020, 021*, *023*, *033*, *035*, *038*, *050*, *077*, *094*, *097*, *105*, and *109*) were highly expressed at all time points during the drought stress. Expression levels of *SiERF002, SiERF003, SiERF016,* and *SiERF109* were maximum at 3 d after drought stress initiation. The expression levels of *SiERF021*, *SiERF023*, *SiERF069*, *SiERF077*, and *SiERF097* were peaked at 7 d, and those of *SiERF005* and *SiERF050* at 10 d, implying their role in the sesame responses to drought stress at different times. Besides, some *SiERF* genes in the down-regulated group such as *SiERF010*, *SiERF014*, *SiERF053*, *SiERF055*, *SiERF078*, *and SiERF093* exhibited a high expression at 3 d. *SiERF11*, *SiERF34*, and *SiERF35* were down-regulated significantly at each time point (Fig. 4A).

Three groups of *SiERF* genes could be distinguished under waterlogging stress (Fig. 4B). The first group constituted of 26 genes that were expressed highly at different time points. Among them, the expression of *SiERF31* and *SiERF54* were significantly up-regulated along with the waterlogging stress progress, indicating they might be essential for sesame survival under waterlogging conditions. The second group of *SiERF* genes (51 genes) was up-regulated at one, two, or three time points, except for *SiERF010*, *SiERF053*, *SiERF011*, and *SiERF081,* which were down-regulated at each time point. The third group of *SiERF* genes was composed of 24 genes that were expressed weakly under waterlogging stress. By integrating the results, we found that twenty-two *SiERF* genes, including *SiERF23*, *SiERF35*, and *SiERF54,* were up-regulated significantly at least once under drought and waterlogging stresses*.* Forty-two *SiERF* genes exhibited contradictory expression patterns under drought and waterlogging stress. For example, *SiERF005*, *SiERF021*, *SiERF38*, *SiERF40*, *SiERF069*, *SiERF98*, *SiERF105*, *SiERF109*, and *SiERF113* were up-regulated significantly under drought and down-regulated under waterlogging, while *SiERF088* was induced by waterlogging and repressed by drought.

### Expression profiles of *SiERF* genes in response to osmotic and submerge stresses

**Fig. 5:**
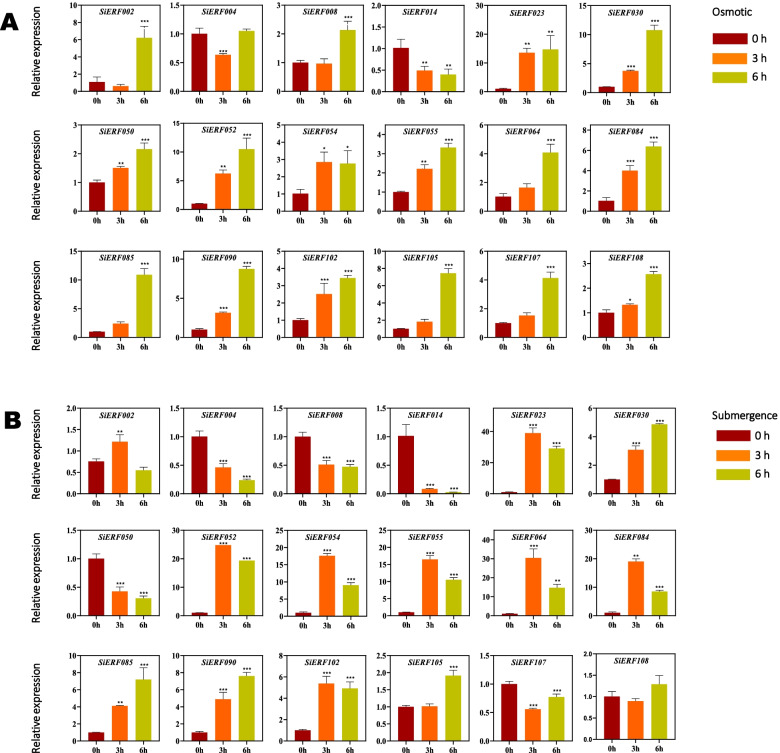
Expression profiles of eighteen *SiERF* genes in sesame leave subjected to osmotic stress (**A**) and submerge stress (**B**) for 6 h. Transcript abundance was quantified using quantitative real-time polymerase chain reaction (qRT-PCR), and expression levels were normalized using sesame *Histone H3.3* (*LOC105159325*) as a reference gene. The mean expression levels from three independent biological replicates were analyzed for significance using t-tests (*p* < 0.01). Asterisks indicate significant expression differences

To further investigate the potential roles of the *SiERF* gene family in response to multiple abiotic stresses in sesame, we selected and examined the stimulation response of eighteen *SiERF* genes under osmotic and submerge stresses via qRT-PCR (Fig. 5A and B). The results showed that except for *SiERF004* and *SiERF014,* the other sixteen *SiERF* genes were significantly up-regulated by osmotic stress, with *SiERF023* exhibiting the highest expression level (Fig. 5A). *SiERF014* was significantly down-regulated, while *SiERF004* expression was not significantly influenced at 6 h. *SiERF023* and *SiERF054* showed a steady tendency of expression profiles from 3 h (Fig. 5A). In contrast to osmotic stress, submerge stress significantly affected the expression of the selected eighteen *SiERF* genes except for *SiERF002* and *SiERF108* (Fig. 5B). *SiERF004*, *SiERF008*, *SiERF014*, *SiERF050* and *SiERF107* were significantly down-regulated while *SiERF023*, *SiERF030*, *SiERF052*, *SiERF054*, *SiERF055*, *SiERF064*, *SiERF084*, *SiERF085*, *SiERF090*, *SiERF102*, and *SiERF105* were significantly up-regulated under the submerge stress (Fig. 5B).

### Expression profiles of *SiERF* genes in response to ABA and ACC treatments

**Fig. 6:**
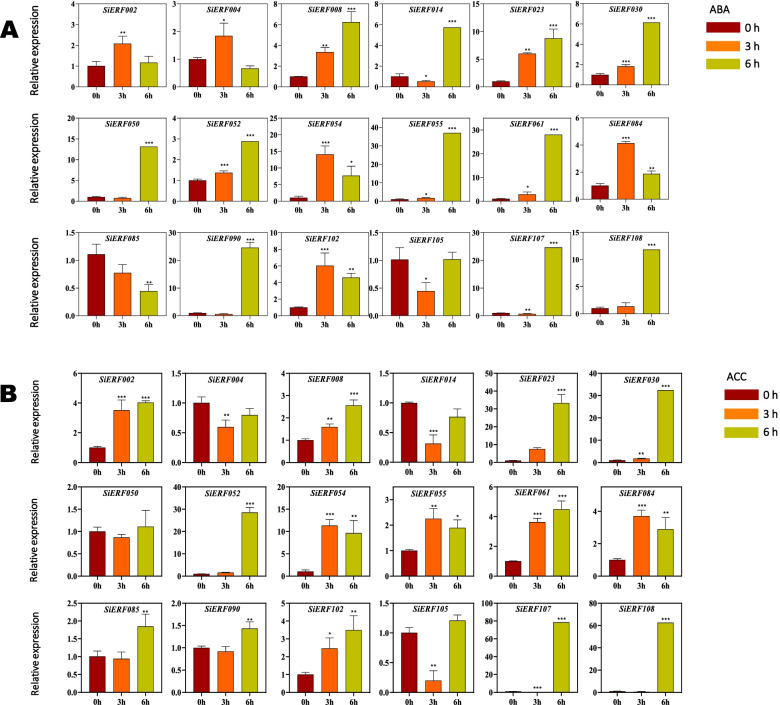
Expression profiles of eighteen *SiERF* genes in sesame leave treated with ABA (**A**) and ACC (**B**) for 6 h. Transcript abundance was quantified using quantitative real-time polymerase chain reaction (qRT-PCR), and expression levels were normalized using sesame *Histone H3.3* (*LOC105159325*) as a reference gene. The mean expression levels from three independent biological replicates were analyzed for significance using t-tests (*p* < 0.01). Asterisks indicate significant expression differences

Abscisic acid (ABA) is a critical plant hormone involved in various growth, developmental, as well as plant and environment interactions processes [45]. 1-aminocyclopropane-1-carboxylic acid (ACC) is the direct precursor of ethylene. It is converted into ethylene in seed plants by ACC oxidase [46]. Ethylene responses in plants are often induced via ACC treatment [47]. We investigated the expression profiles of eighteen selected *SiERF* genes in response to ABA and ACC treatment of sesame for 0 h, 3 h, and 6 h through qRT-PCR. As presented in Fig. 6A and B, the selected *SiERF* genes were up-regulated by both ABA and ACC treatments except for *SiERF004, SiERF014*, *SiERF050*, and *SiERF085*. *SiERF105* was down-regulated by both ABA and ACC treatment. *SiERF050* expression was induced by ABA treatment but was not significantly affected by ACC treatment. *SiERF004* was up- and down-regulated by ABA and ACC, respectively. In contrast, *SiERF085* was down- and up-regulated by ABA and ACC, respectively. The expression of *SiERF023*, *SiERF030*, *SiERF052*, *SiERF055*, *SiERF061*, and *SiERF107* were significantly induced along with the duration of the ABA treatment, specifically at 6 h (Fig. 6A). Meanwhile, the same genes with *SiERF002*, *SiERF008,* and *SiERF102* exhibited the same expression patterns under ACC (Fig. 6B).

## Discussion

Sesame is one of the most important oilseed crops supplying humans worldwide with various metabolites, including high-quality nutrients and bioactive compounds [1, 7]. The plant growth, development, survival, reproduction, and yield are usually affected by various abiotic stresses [10–12, 16]. To adapt to unfavorable environmental conditions, the plant has implemented sophisticated regulatory mechanisms involving diverse TFs [10, 48]. Among them, ERF genes have emerged as one of the key regulators of multiple stress responses in sesame [16, 17]. Therefore, in this study, we performed a comprehensive and systematic analysis of the ERF gene family in sesame and investigated the expression of *SiERFs* under various abiotic stresses.

The ERF gene family represents one of the largest families of plant TFs and is essential for plant species survival [23]. ERF genes have been widely identified in many plants, including *Arabidopsis*, rice, soybean, *Brassica napus*, *Sorghum bicolor*, Tartary buckwheat, *Medicago sativa*, and peanuts in which 122, 139, 323, 444, 158, 116, 159, and 63 *ERFs* were detected, respectively [22, 23, 28, 30, 49–51]. Herein, we identified 114 *SiERFs*, indicating that the ERF gene family has expanded more in many species compared with *S. indicum*. A similar observation was noticed by Dossa et al. [20]. The *SiERFs* were distributed irregularly on the sixteen LGs of the sesame genome, mostly in a cluster of two or three genes. It is shown that a subset of the ERF genes appears in clusters on the chromosomes and contributes together to regulate metabolism [51]. The interspecific variation of the number of ERF genes may be originated from differences in gene duplication events. Studies revealed that the expansion of the ERF gene family in plants might be caused by chromosomal (segmental) duplication and tandem duplication [22, 30]. We found that the *SiERF* gene family went through whole-genome duplication (WGD) and tandem duplication events. 52.73% of the *SiERFs* were derived from WGD events, indicating that WGD is essential for ERF gene family expansion in sesame.

78.94% of the *SiERF* genes were intron-less and contained one exon. Meanwhile, 20 *SiERF* genes were constituted of two exons and one intron. 60% and 38 *SbERFs* also had no and single intron, respectively [50]. Also, the 40 identified *cis*-acting elements in the promoter regions of 114 *SiERFs* were related to light-response, stress-response, and hormone response. These results suggest that *SiERFs* might play essential roles during the sesame plant growth, development, and reproduction. Particularly, *SiERFs* might exhibit efficient expression in swift response to environmental stresses. Phylogenetic analysis showed that *SiERF* family proteins were systematically classified into 11 subgroups as the previously classified *AtERFs* by Nakano et al., except for the group Xb-L [22]. The ERF genes in *S. bicolor* and *Hypericum perforatum* were similarly classified in 11 groups [24, 50]. The motif analysis showed that *SiERFs* in the same clade shared a similar motif structuring, indicating the reliability of the phylogenetic classification of the ERF proteins and the coevolution of the ERF domain with the remaining protein sequence. Most of the *SiERFs* conserved motifs 1–4, suggesting they might be involved in a regulation network of developmental processes and abiotic stresses response in sesame. In *Arabidopsis*, studies demonstrated that AP2/ERFs participate in various stress tolerance, allowing them to build an interconnected stress regulatory network [52]. Some motifs were specific to phylogenetic groups suggesting their potential contribution to the *SiERF* gene’s functional specialization. Taken together, these findings denote that *SiERFs* within the same subgroups could play similar functions. These functions could be predicted based on the reported roles of the *Arabidopsis ERF* genes. Indeed, it was shown that the sequences gathered in the same clade play similar physiological functions [53]. For example, *GmERF135* and *OsERF922* in soybean and rice, respectively, and their homologous maize *ZmERF39* and *ZmERF23* were both up-regulated by drought and salt stress [38, 54]. The *A. thaliana* ERF-VII group plays an important role in low-oxygen sensing and low-oxygen survival and root growth [55, 56]. Therefore, we speculated that the *SiERF* genes belonging to group VII might be involved in hypoxia response and root development [57, 58].

The sustainability of crop production requires an in-depth understanding of the stress-induced molecular mechanisms in plants and the identification of multiple stress-responsive candidate genes for targeted improvement of crop tolerance to unfavorable growth conditions. Previous studies in sesame, *Arabidopsis*, *Panax ginseng*, *Triticum durum*, etc., showed evidence that ERF TFs are essential for plant response to abiotic stresses [16, 17, 59–61]. Wan et al. reported that ectopic overexpression of the peanuts *AhERF019* improved tolerance to drought, salt, and heat stresses in *Arabidopsis* [28]. Overexpression of *AtERF1*, *AtERF019*, *AtERF71*, and *AtERF98* enhanced the *Arabidopsis* plant tolerance to drought, heat, salt, and osmotic stresses [33–36]. We then investigated the expression of *SiERF* genes under drought and waterlogging stress. We found that 59 and 26 *SiERFs* were significantly induced under drought and waterlogging stress, respectively, confirming their pivotal role in drought and waterlogging stresses tolerance in sesame. The up-regulated *SiERF* genes reached their expression peak at different time points, indicating they might be involved in different stress-responsive processes. Moreover, the qRT-PCR analysis revealed that most of the *SiERFs* that responded to the drought and waterlogging stresses were also induced significantly under osmotic, submerge, ABA, and ACC (an immediate precursor of ethylene) treatments. Among them, *SiERF23* and *SiERF54* were the most induced by both the abiotic stresses. ABA and ethylene play essential roles in various plant growth and developmental processes, including seed maturation, germination, abiotic stress responses, pathogen response, senescence, etc. [9, 62, 63]. These findings support that the ERF gene family plays a vital role during sesame growth and development, especially in the plant responses to abiotic stresses. In addition, they suggest that targeting *SiERF23* and *SiERF54* could help promote sesame tolerance to multiple abiotic stresses.

## Conclusion

In this study, 114 *SiERF* genes were identified and comprehensively analyzed. Chromosomal locations, phylogenetic relationships, gene structures, conserved motifs, and cis-acting elements analyses revealed that *SiERFs* might be involved in networks regulation of various developmental processes, especially in stresses tolerance in sesame. Tandem duplication and mostly whole-genome duplication are the driving forces that have contributed to the ERF gene family expansion in sesame. Gene expression profiles and qRT-PCR analyses unveiled that many *SiERFs* are stimulated under drought, waterlogging, osmotic, and submerge stresses. Particularly, *SiERF23* and *SiERF54* were identified as potential candidate genes for targeted improvement of multiple abiotic stresses tolerance in sesame. This study provides reference information for exploring the *SiERF* gene’s functions and investigating the regulatory mechanisms involved in abiotic stresses resistance in sesame.

## Materials and methods

### Plant material

The sesame variety Zhongzhi No. 13 used in this study was provided by the Oil Crops Research Institute of the Chinese Academy of Agricultural Science (OCRI-CAAS, Wuhan, China).

### Identification of ERF family genes in the sesame genome

Whole-genome protein sequences of *Sesamum indicum* were downloaded from NCBI (https://ftp.ncbi.nlm.nih.gov/genomes/refseq/plant/Sesamum_indicum/latest_assembly_versions/GCF_000512975.1_S_indicum_v1.0/). A local BLASTP alignment against all sesame proteins was established by using known ERF protein sequences from *Arabidopsis* as queries with a cut-off e-value of 1E-10. The Hidden Markov Model (HMM) profile of the AP2 domain (PF00847) and the B3 domain (PF02362) were downloaded from the PFAM database (http://pfam.xfam.org/) [64], and used to search against the sesame protein sequences using HMMER3.0 [65], with a threshold of E < 1E-4. The presence of the AP2 domain in the putative sesame ERF proteins was further confirmed by SMART (http://smart.embl-heidelberg.de/) [66]. After removed the proteins containing two repeated AP2 domains or B3 domains, the remaining proteins were assigned as members of the ERF family in sesame.

### Chromosomal localization and gene duplication analyses

All identified ERF genes were mapped to the sesame linkage groups based on positions information using TBtools software [67]. Gene duplication analyses were performed using the One-Step MCScanX function in TBtools software, and the result was further visualized by the Circle Gene View function [67]. Genes that were located on the unassembled genomic scaffolds were excluded from analyses.

### Multiple sequence alignment and phylogenetic analysis

Multiple sequence alignment of ERF proteins from sesame and *Arabidopsis* was performed using Clustal X [68]. Subsequently, an unrooted phylogenetic tree with 1000 bootstrap replications was constructed by the MEGA (version 5.0) program [69] using the neighbor-joining (NJ) method and based on the conserved AP2/ERF domain of ERFs from sesame and *Arabidopsis*.

### Gene structure, conserved motifs, and *cis*-acting elements analyses

The gene structure of *SiERFs* was analyzed by TBtools software [67] based on gene’s structure annotation file in GFF3 format of sesame. Conserved motifs of *SiERFs* were analyzed using MEME (Multiple Em for Motif Elicitation) v5.3.3 (http://meme-suite.org/tools/meme) [70] with the default parameters. The XML file storing motif pattern information obtained from MEME was used to generate schematic diagrams of motif distribution by TBtools software [67].

To analyze the *cis*-acting elements in the promoter region, the 1500-bp length of the upstream DNA sequences of *SiERF* genes were extracted in TBtools software and submitted to the PlantCARE database (http://bioinformatics.psb.ugent.be/webtools/plantcare/html/) [43].

### Expression profiling of *SiERF* genes under drought and waterlogging

The expression levels of *SiERF* genes in response to drought and waterlogging stress were analyzed using the RNA-seq data previously developed by our group [9, 44]. The heatmap was constructed by TBtools software with Log2-based expression fold-changes [67]. The differentially expressed genes (DEGs) were identified at the criteria of false discovery rate (FDR) < 0.01 and |log2FC (fold change)| > 1.

### Osmotic, submerge, ABA, and ACC treatments

The Zhongzhi No. 13 seeds were grown in a growth chamber at 28 °C (16 h light/8 h dark cycle). The different treatments were induced on two-week-old seedlings. The osmotic stress was induced as described in our previous study [71]. For the submerge stress, the seedlings were introduced into distilled water at a depth of 3 cm from the water surface. The hormone treatments were performed as per Yin et al. [72]. 0.1 mM ABA and ACC were sprayed on the surface of the seedling leaves. The leaf samples were collected after each treatment at 0 h, 3 h, and 6 h for genes expression analysis. All collected samples were frozen immediately in liquid nitrogen and stored at − 80 °C until use.

### qRT-PCR

Total RNA was isolated from each sample, and first-strand cDNAs were synthesized following the methods reported by Wei et al. [73]. Quantitative real-time PCR (qRT-PCR) was performed in Roche LightCycler 480 real-time PCR system with the ChamQ SYBR qPCR Master Mix (Vazyme Biotech, China). The experiment was performed with three replicates. Relative expression levels were calculated according to the 2^–ΔΔCT^ method and normalized to the sesame *Histone H3.3* (*LOC105159325*) gene expression [71, 74]. The gene-specific primers are listed in Table S5.

## Supplementary Information

**Additional file 1: Table S1.** Detailed information of *Sesamun indicum* ERF (*SiERF*) genes; **Table S2.** Sequence Characteristics of *SiERF* genes; **Table S3.** Number of each *cis*-acting element in the promoter region of SiERF genes; **Table S4.** Information related to the *cis*-acting elements identified in the *SiERF* genes; **Table S5.** List of primers used for the qRT-PCR analysis.

**Additional file 2: Fig. S1.** Ortholog and duplication analysis of *SiERF* genes; **Fig. S2.** The logos of 16 conserved motifs in *SiERF* proteins; **Fig. S3.** Distribution of *cis*-acting elements in the promoter regions of the SiERFs. The number of *SiERF* genes containing each *cis*-acting element.

## Data Availability

The datasets generated and/or analysed (whole-genome protein sequences of sesame) during the current study are available in the NCBI repository (https://ftp.ncbi.nlm.nih.gov/genomes/refseq/plant/Sesamum_indicum/latest_assembly_versions/GCF_000512975.1_S_indicum_v1.0/). All data generated or analysed during this study are included in this published article and its supplementary information files.

## Abbreviations

AP2/ERF: APETALA2/Ethylene-Responsive Factor
SiERF: Sesamum indicum ethylene response factor
TF: Transcription factor
qRT-PCR: Quantitative real-time PCR
BLASTP: Basic Local Alignment Search Tool
HMM: Hidden Markov model
MW: Molecular weight
pI: Theoretical isoelectric point
II: The instability index
GRAVY: Aliphatic index and grand average of hydropathicity
ABA: Abscisic acid
ACC: 1-aminocyclopropane-1-carboxylic acid

## References

[1] Niti P, K RA, Ratna K, V BK (2014). Value addition in sesame: a perspective on bioactive components for enhancing utility and profitability. Pharmacogn Rev.

[2] Kim A-Y, Yun C-I, Lee J-G, Kim Y-J (2020). Determination and daily intake estimation of Lignans in sesame seeds and sesame oil products in Korea. Foods.

[3] Oyinloye B, Ajiboye B, Ojo O, Nwozo S, Kappo A (2016). Cardioprotective and antioxidant influence of aqueous extracts from Sesamum indicum seeds on oxidative stress induced by cadmium in wistar rats. Pharmacogn Mag.

[4] Patel A, Bahna SL (2016). Hypersensitivities to sesame and other common edible seeds. Allergy.

[5] Namiki M (2007). Nutraceutical functions of sesame: a review. Crit Rev Food Sci Nutr.

[6] F MA, Mariam M, K NG (2017). A comprehensive review on the anti-cancer properties and mechanisms of action of sesamin, a lignan in sesame seeds (Sesamum indicum). Eur J Pharmacol.

[7] Mebeaselassie A, Maria V, Anna R, Evelyn M, Petr K (2021). Lignans of sesame (Sesamum indicum L.): a comprehensive review. Molecules.

[8] Kermani SG, Saeidi G, Sabzalian MR, Gianinetti A (2019). Drought stress influenced sesamin and sesamolin content and polyphenolic components in sesame ( Sesamum indicum L.) populations with contrasting seed coat colors. Food Chem.

[9] Dossa K, You J, Wang L, Zhang Y, Li D, Zhou R, Yu J, Wei X, Zhu X, Jiang S (2019). Transcriptomic profiling of sesame during waterlogging and recovery. Sci Data.

[10] Wang L, Li D, Zhang Y, Gao Y, Yu J, Wei X, Zhang X (2016). Tolerant and susceptible sesame genotypes reveal waterlogging stress response patterns. PLoS One.

[11] Komivi D, Donghua L, Linhai W, Xiaomin Z, Jingyin Y, Xin W, Daniel F, Diaga D, Boshou L, Ndiaga C (2017). Dynamic transcriptome landscape of sesame ( Sesamum indicum L.) under progressive drought and after rewatering. Genom Data.

[12] Anee TI, Nahar K, Rahman A, Mahmud JA, Bhuiyan TF, Alam MU, Fujita M, Hasanuzzaman M (2019). Oxidative damage and antioxidant defense in Sesamum indicum after different waterlogging durations. Plants.

[13] Sardar-Ali K, Meng-Zhan L, Suo-Min W, Hong-Ju Y (2018). Revisiting the role of plant transcription factors in the battle against abiotic stress. Int J Mol Sci.

[14] Baillo EH, Kimotho RN, Zhang Z, Xu P (2019). Transcription factors associated with abiotic and biotic stress tolerance and their potential for crops improvement. Genes.

[15] M HA, Narendra T (2013). Biotech crops: imperative for achieving the millenium development goals and sustainability of agriculture in the climate change era. GM Crops Food.

[16] Dossa K, Mmadi MA, Zhou R, Zhang T, Su R, Zhang Y, Wang L, You J, Zhang X (2019). Depicting the Core transcriptome modulating multiple abiotic stresses responses in sesame (Sesamum indicum L.). Int J Mol Sci.

[17] Linhai W, Komivi D, Jun Y, Yanxin Z, Donghua L, Rong Z, Jingyin Y, Xin W, Xiaodong Z, Shiyang J (2020). High-resolution temporal transcriptome sequencing unravels ERF and WRKY as the master players in the regulatory networks underlying sesame responses to waterlogging and recovery. Genomics.

[18] Mmadi M, Dossa K, Wang L, Zhou R, Wang Y, Cisse N, Sy M, Zhang X (2017). Functional characterization of the versatile MYB gene family uncovered their important roles in plant development and responses to drought and waterlogging in sesame. Genes (Basel).

[19] Donghua L, Pan L, Jingyin Y, Linhai W, Komivi D, Yanxin Z, Rong Z, Xin W, Xiurong Z (2017). Genome-wide analysis of WRKY gene family in the sesame genome and identification of the WRKY genes involved in responses to abiotic stresses. BMC Plant Biol.

[20] Komivi D (2016). Insight into the AP2/ERF transcription factor superfamily in sesame and expression profiling of DREB subfamily under drought stress. BMC Plant Biol.

[21] Yoh S, Qiang L, G DJ, Hiroshi A, Kazuo S, Kazuko Y (2002). DNA-binding specificity of the ERF/AP2 domain of Arabidopsis DREBs, transcription factors involved in dehydration- and cold-inducible gene expression. Biochem Biophys Res Commun.

[22] Toshitsugu N, Kaoru S, Tatsuhito F, Hideaki S (2006). Genome-wide analysis of the ERF gene family in Arabidopsis and Rice. Plant Physiol.

[23] Xiaoyu J, Xiaofan Y, Boniface N, Zhengshe Z, Xueyang M, Xiaoshan L, Yanrong W, Wenxian L (2019). Genome-wide identification and expression profiling of the ERF gene family in Medicago sativa L. under various abiotic stresses. DNA Cell Biol.

[24] Qian Z, Wen Z, Bin L, Lin L, Meng F, Li Z, Xiaoding Y, Donghao W, Zhezhi W (2021). Genome-wide analysis and the expression pattern of the ERF gene family in Hypericum perforatum. Plants.

[25] Most SA, Mohammed N, Kouji S, Takumi S, Hiroaki K, Takahide S, Il-Ryong C, Toshihiro O, Shoshi K (2011). Gene structures, classification and expression models of the AP2/EREBP transcription factor family in rice. Plant Cell Physiol.

[26] Zhang G, Chen M, Chen X, Xu Z, Guan S, Li L-C, Li A, Guo J, Mao L, Ma Y (2008). Phylogeny, gene structures, and expression patterns of the ERF gene family in soybean ( Glycine max L.). J Exp Bot.

[27] Sharma MK, Kumar R, Solanke AU, Sharma R, Tyagi AK, Sharma AK (2010). Identification, phylogeny, and transcript profiling of ERF family genes during development and abiotic stress treatments in tomato. Mol Gen Genomics.

[28] Wan L, Wu Y, Huang J, Dai X, Lei Y, Yan L, Jiang H, Zhang J, Varshney RK, Liao B (2014). Identification of ERF genes in peanuts and functional analysis of AhERF008 and AhERF019 in abiotic stress response. Funct Integr Genomics.

[29] Zhou M-L, Tang Y-X, Wu Y-M (2012). Genome-wide analysis of AP2/ERF transcription factor family in Zea Mays. Curr Bioinforma.

[30] Ghorbani R, Zakipour Z, Alemzadeh A, Razi H (2020). Genome-wide analysis of AP2/ERF transcription factors family in Brassica napus. Physiol Mol Biol Plants.

[31] Waheed RM, Jie L, Liaqat S, Liu Y, Can C, Dong MX, Liu X, Aamir MM, Muhammad A, Shamsur R (2021). Expansion and molecular characterization of AP2/ERF gene family in wheat (Triticum aestivum L.). Front Genet.

[32] Debbarma J, Sarki YN, Saikia B, Boruah HPD, Singha DL, Chikkaputtaiah C (2019). Ethylene response factor (ERF) family proteins in abiotic stresses and CRISPR–Cas9 genome editing of ERFs for multiple abiotic stress tolerance in crop plants: a review. Mol Biotechnol.

[33] Cheng M-C, Liao P-M, Kuo W-W, Lin T-P (2013). The Arabidopsis ETHYLENE RESPONSE FACTOR1 regulates abiotic stress-responsive gene expression by binding to different cis-acting elements in response to different stress signals. Plant Physiol.

[34] Scarpeci TE, Frea VS, Zanor MI, Valle EM (2017). Overexpression of AtERF019 delays plant growth and senescence, and improves drought tolerance in Arabidopsis. J Exp Bot.

[35] Park H-Y, Seok H-Y, Woo D-H, Lee S-Y, Tarte VN, Lee E-H, Lee C-H, Moon Y-H (2011). AtERF71/HRE2 transcription factor mediates osmotic stress response as well as hypoxia response in Arabidopsis. Biochem Biophys Res Commun.

[36] Zhang Z, Wang J, Zhang R, Huang R (2012). The ethylene response factor AtERF98 enhances tolerance to salt through the transcriptional activation of ascorbic acid synthesis in Arabidopsis. Plant J.

[37] Dong-Keun L, Harin J, Geupil J, Seo JJ, Shic KY, Sun-Hwa H, Yang DC, Ju-Kon K (2016). Overexpression of the OsERF71 transcription factor alters Rice root structure and drought resistance. Plant Physiol.

[38] Liu D, Chen X, Liu J, Ye J, Guo Z (2012). The rice ERF transcription factor OsERF922 negatively regulates resistance to Magnaporthe oryzae and salt tolerance. J Exp Bot.

[39] Zhang G, Chen M, Li L, Xu Z, Chen X, Guo J, Ma Y (2009). Overexpression of the soybean GmERF3 gene, an AP2/ERF type transcription factor for increased tolerances to salt, drought, and diseases in transgenic tobacco. J Exp Bot.

[40] Ma L, Hu L, Fan J, Amombo E, Khaldun ABM, Zheng Y, Chen L (2017). Cotton GhERF38 gene is involved in plant response to salt/drought and ABA. Ecotoxicology.

[41] Wenbin L, Yayun L, Yiling Y, Gan W, Ming P (2016). Exposure to various abscission-promoting treatments suggests substantial ERF subfamily transcription factors involvement in the regulation of cassava leaf abscission. BMC Genomics.

[42] Yao W, An T, Xu Z, Zhang L, Gao H, Sun W, Liao B, Jiang C, Liu Z, Duan L (2020). Genomic-wide identification and expression analysis of AP2/ERF transcription factors related to andrographolide biosynthesis in Andrographis paniculata. Industr Crops Prod.

[43] Ren A, Ahmed RI, Chen HY, Han LH, Sun JH, Ding AM, Guo YF, Kong YZ (2019). Genome-wide identification, characterization and expression patterns of the pectin Methylesterase inhibitor genes in Sorghum bicolor. Genes.

[44] You J, Zhang Y, Liu A, Li D, Wang X, Dossa K, Zhou R, Yu J, Zhang Y, Wang L (2019). Transcriptomic and metabolomic profiling of drought-tolerant and susceptible sesame genotypes in response to drought stress. BMC Plant Biol.

[45] Finkelstein R (2013). Abscisic acid synthesis and response. Arabidopsis Book.

[46] Dongdong L, Eduardo FS, Uzair A, Andrew C, CJ M, BJ L, Caren C (2020). Ethylene-independent functions of the ethylene precursor ACC in Marchantia polymorpha. Nat Plants.

[47] Mou W, Kao Y-T, Michard E, Simon AA, Li D, Wudick MM, Lizzio MA, Feijó JA, Chang C (2020). Ethylene-independent signaling by the ethylene precursor ACC in Arabidopsis ovular pollen tube attraction. Nature. Nat Commun.

[48] Dossa K, Li D, Wang L, Zheng X, Liu A, Yu J, Wei X, Zhou R, Fonceka D, Diouf D (2017). Transcriptomic, biochemical and physio-anatomical investigations shed more light on responses to drought stress in two contrasting sesame genotypes. Sci Rep.

[49] Wenbo J, Xuejing Z, Xuewei S, Junfeng Y, Yongzhen P (2020). Genome-wide identification and characterization of APETALA2/ethylene-responsive element binding factor superfamily genes in soybean seed development. Front Plant Sci.

[50] Mathur S, Priyadarshini SS, Singh V, Vashisht I, Jung K-H, Sharma R, Sharma MK (2020). Comprehensive phylogenomic analysis of ERF genes in sorghum provides clues to the evolution of gene functions and redundancy among gene family members. 3 Biotech.

[51] Liu M, Sun W, Ma Z, Zheng T, Huang L, Wu Q, Zhao G, Tang Z, Bu T, Li C (2019). Genome-wide investigation of the AP2/ERF gene family in tartary buckwheat (Fagopyum Tataricum). BioMed Central.

[52] Yin D, Sun D, Han Z, Ni D, Norris A, Jiang C-Z (2019). PhERF2 , an ethylene-responsive element binding factor, plays an essential role in waterlogging tolerance of petunia. Hortic Res.

[53] GD J, Vicente CJ, Sophie B, Geeta P, MG M, HM J (2015). Group VII ethylene response factors coordinate oxygen and nitric oxide signal transduction and stress responses in plants. Plant Physiol.

[54] Zhao M-J, Yin L-J, Ma J, Zheng J-C, Wang Y-X, Lan J-H, Fu J-D, Chen M, Xu Z-S, Xu Z-S (2019). The Roles of GmERF135 in Improving Salt Tolerance and Decreasing ABA Sensitivity in Soybean. Front Plant Sci.

[55] Zhao M-J, Yin L-J, Liu Y, Ma J, Zheng J-C, Lan J-H, Fu J-D, Chen M, Xu Z-S, Ma Y-Z (2019). The ABA-induced soybean ERF transcription factor gene GmERF75 plays a role in enhancing osmotic stress tolerance in Arabidopsis and soybean. BMC Plant Biol.

[56] Gasch P, Fundinger M, Müller JT, Lee T, Bailey-Serres J, Mustroph A (2016). Redundant ERF-VII transcription factors bind to an evolutionarily conserved cis-motif to regulate hypoxia-responsive gene expression in Arabidopsis. Plant Cell.

[57] HyeYeon S, Jimin H, SunYoung L, Hyoungjoon B, YongHwan M (2020). Two alternative splicing variants of AtERF73/HRE1, HRE1α and HRE1β, have differential transactivation activities in Arabidopsis. Int J Mol Sci.

[58] Yang C-Y, Huang Y-C, Ou S-L (2017). ERF73/HRE1 is involved in H_2_O_2_ production via hypoxia-inducible Rboh gene expression in hypoxia signaling. Protoplasma.

[59] Shoji T, Yuan L (2020). ERF gene clusters: working together to regulate metabolism. Trends Plant Sci.

[60] Jing C, Yuanhang Z, Qi Z, Qian L, Li L, Chunyu S, Kangyu W, Yanfang W, Mingzhu Z, Hongjie L (2020). Structural variation, functional differentiation and expression characteristics of the AP2/ERF gene family and its response to cold stress and methyl jasmonate in Panax ginseng C.A. Meyer. PLoS One.

[61] Sahar F, Ertugrul F, Kamal KS, Alessandro V, Fabio P, Gianni B, Parviz H (2020). The AP2/ERF gene family in Triticum durum: genome-wide identification and expression analysis under drought and salinity stresses. Genes.

[62] Nascimento FX, Rossi MJ, Soares CRFS, McConkey BJ, Glick BR (2017). New insights into 1-aminocyclopropane-1-carboxylate (ACC) deaminase phylogeny, evolution and ecological significance. PLoS One.

[63] Van de Poel B, Van Der Straeten D (2014). 1-aminocyclopropane-1-carboxylic acid (ACC) in plants: more than just the precursor of ethylene!. Front Plant Sci.

[64] Mistry J, Chuguransky S, Williams L, Qureshi M, Salazar Gustavo A, Sonnhammer ELL, Tosatto SCE, Paladin L, Raj S, Richardson LJ (2021). Pfam: the protein families database in 2021. Nucleic Acids Res.

[65] Wheeler TJ, Eddy SR (2013). Nhmmer: DNA homology search with profile HMMs. Bioinformatics.

[66] Letunic I, Khedkar S, Bork P (2021). SMART: recent updates, new developments and status in 2020. Nucleic Acids Res.

[67] Chen C, Chen H, Zhang Y, Thomas HR, Frank MH, He Y, Xia R (2020). TBtools: An integrative toolkit developed for interactive analyses of big biological data. Mol Plant.

[68] Thompson JD, Gibson TJ, Plewniak F, Jeanmougin F, Higgins DG (1997). The CLUSTAL_X windows interface: flexible strategies for multiple sequence alignment aided by quality analysis tools. Nucleic Acids Res.

[69] Tamura K, Peterson D, Peterson N, Stecher G, Nei M, Kumar S (2011). MEGA5: molecular evolutionary genetics analysis using maximum likelihood, evolutionary distance, and maximum parsimony methods. Mol Biol Evol.

[70] Bailey TL, Boden M, Buske FA, Frith M, Grant CE, Clementi L, Ren J, Li WW, Noble WS (2009). MEME SUITE: tools for motif discovery and searching. Nucleic Acids Res.

[71] You J, Wang Y, Zhang Y, Dossa K, Li D, Zhou R, Wang L, Zhang X (2018). Genome-wide identification and expression analyses of genes involved in raffinose accumulation in sesame. Sci Rep.

[72] Lili Y, Meiling Z, Ruigang W, Xiaoliang C, Fei L, Baolong X (2021). Genome-wide analysis of OSCA gene family members in Vigna radiata and their involvement in the osmotic response. BMC Plant Biol.

[73] Wei M, Liu A, Zhang Y, Zhou Y, Li D, Dossa K, Zhou R, Zhang X, You J (2019). Genome-wide characterization and expression analysis of the HD-zip gene family in response to drought and salinity stresses in sesame. BMC Genomics.

[74] Livak KJ, Schmittgen TD (2001). Analysis of relative gene expression data using real-time quantitative PCR and the 2(−Delta Delta C(T)) method. Methods.

