# Correction: Genome-wide characterization and identification of candidate *ERF* genes involved in various abiotic stress responses in sesame (*Sesamum indicum* L.)

**DOI:** 10.1186/s12870-022-03666-x

**Published:** 2022-06-02

**Authors:** Ruqi Su, Senouwa Segla Koffi Dossou, Komivi Dossa, Rong Zhou, Aili Liu, Yanping Zhong, Sheng Fang, Xiurong Zhang, Ziming Wu, Jun You

**Affiliations:** 1 Key Laboratory of Crop Physiology, Ecology, and Genetic Breeding, Ministry of Education/College of Agronomy, Jiangxi Agricultural University, Nanchang, 330045 China; 2 Key Laboratory of Biology and Genetic Improvement of Oil Crops of the Ministry of Agriculture and Rural Affairs, Oil Crops Research Institute of the Chinese Academy of Agricultural Sciences, Wuhan, 430062 China; 3 CIRAD, UMR AGAP Institut, F-34398 Montpellier, France; 4 UMR AGAP Institut, Univ Montpellier, CIRAD, INRAE, Institut Agro, F-34398 Montpellier, France

**Correction: BMC Plant Biol 22, 256 (2022)**

**https://doi.org/10.1186/s12870-022-03632-7**

Following publication of the original article [1], authors identified an error found in Affiliations 1 and 2. The correct affiliation details are presented below:

^1^Key Laboratory of Crop Physiology, Ecology, and Genetic Breeding, Ministry of Education/College of Agronomy, Jiangxi Agricultural University, Nanchang 330045, China

^2^Key Laboratory of Biology and Genetic Improvement of Oil Crops of the Ministry of Agriculture and Rural Affairs, Oil Crops Research Institute of the Chinese Academy of Agricultural Sciences, Wuhan 430062, China

The corrections do not have any effect on the final conclusions of the paper. The original article has been corrected.

## References

[1] Su R, Dossou SSK, Dossa K (2022). Genome-wide characterization and identification of candidate *ERF* genes involved in various abiotic stress responses in sesame (*Sesamum indicum* L.). BMC Plant Biol.

