# Supplementary material for: Genome-wide characterization and identification of candidate ERF genes involved in various abiotic stress responses in sesame (Sesamum indicum L.)
Source: BMC Plant Biol. 2022 May 24;22:256. doi: 10.1186/s12870-022-03632-7 (PMC9128266; doi:10.1186/s12870-022-03632-7)
Supplement: Supplementary file 2 — Additional file 2: Fig. S1. Ortholog and duplication analysis of SiERF genes; Fig. S2. The logos of 16 conserved motifs in SiERF proteins; Fig. S3. Distribution of cis-acting elements in the promoter regions of the SiERFs. The number of SiERF genes containing each cis-acting element. [file 12870_2022_3632_MOESM2_ESM.pdf]

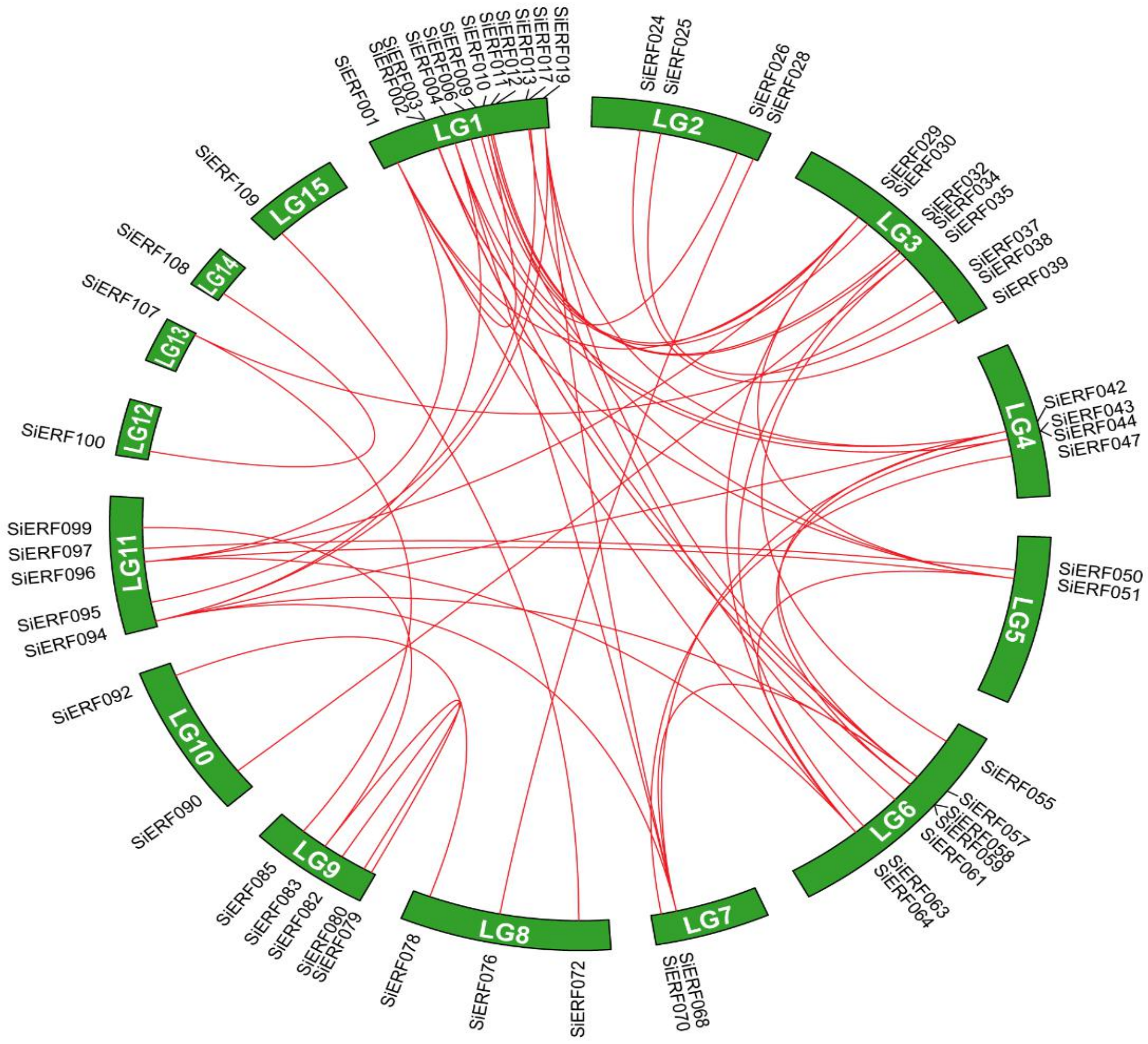

LG1
LG2
LG3
LG4
LG5
LG6
LG7
LG8
LG9
LG10
LG11
LG12
LG13
LG14
LG15
SiERF001
SiERF002
SiERF003
SiERF004
SiERF006
SiERF009
SiERF010
SiERF011
SiERF012
SiERF013
SiERF017
SiERF019
SiERF024
SiERF025
SiERF026
SiERF028
SiERF029
SiERF030
SiERF032
SiERF034
SiERF035
SiERF037
SiERF038
SiERF039
SiERF042
SiERF043
SiERF044
SiERF047
SiERF050
SiERF051
SiERF055
SiERF057
SiERF058
SiERF059
SiERF061
SiERF063
SiERF064
SiERF068
SiERF070
SiERF072
SiERF076
SiERF078
SiERF079
SiERF080
SiERF082
SiERF083
SiERF085
SiERF090
SiERF092
SiERF094
SiERF095
SiERF096
SiERF097
SiERF099
SiERF100
SiERF107
SiERF108
SiERF109

Fig. S1

	Width	Sites	E-value	Logo
Motif 1	21	113	5.8e-1719	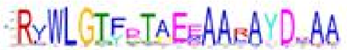
Motif 2	11	109	1.7e-873	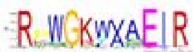
Motif 3	15	106	2.1e-604	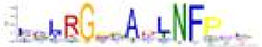
Motif 4	11	105	1.3e-441	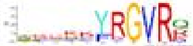
Motif 5	21	24	2.0e-142	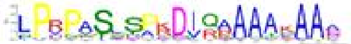
Motif 6	20	30	1.1e-082	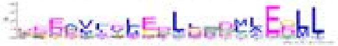
Motif 7	29	11	2.7e-078	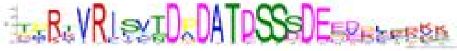
Motif 8	34	8	3.2e-047	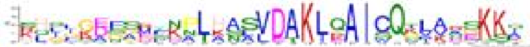
Motif 9	20	7	1.2e-025	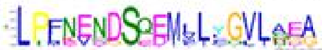
Motif 10	41	4	4.7e-024	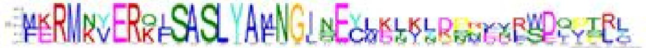
Motif 11	33	6	1.3e-019	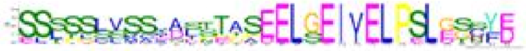
Motif 12	19	7	6.8e-020	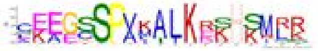
Motif 13	6	27	1.6e-019	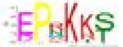
Motif 14	29	4	1.7e-019	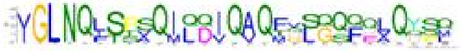
Motif 15	21	4	1.2e-017	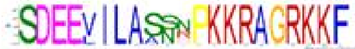
Motif 16	49	2	1.3e-017	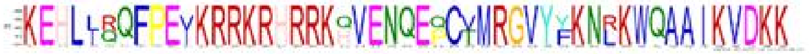

Fig. S2

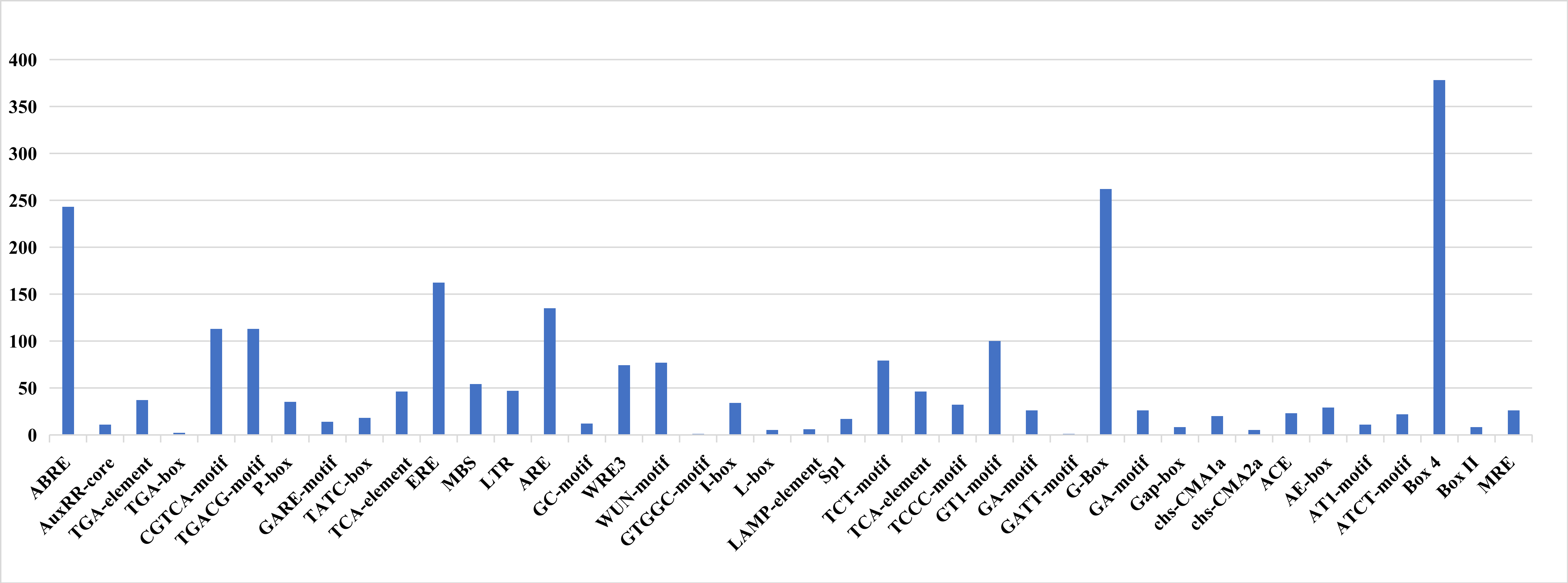

400
350
300
250
200
150
100
50
0
ABRE
AuxRR-core
TGA-element
TGA-box
CGTCA-motif
TGACG-motif
P-box
GARE-motif
TATC-box
TCA-element
ERE
MBS
LTR
ARE
GC-motif
WRE3
WUN-motif
GTGGC-motif
I-box
L-box
LAMP-element
Sp1
TCT-motif
TCA-element
TCCC-motif
GT1-motif
GA-motif
GATT-motif
G-Box
GA-motif
Gap-box
chs-CMA1a
chs-CMA2a
ACE
AE-box
AT1-motif
ATCT-motif
Box 4
Box II
MRE

Fig. S3
